# From metagenomic data to personalized in silico microbiotas: predicting dietary supplements for Crohn’s disease

**DOI:** 10.1038/s41540-018-0063-2

**Published:** 2018-08-01

**Authors:** Eugen Bauer, Ines Thiele

**Affiliations:** Luxembourg Centre for Systems Biomedicine, Universite du Luxembourg, Esch-sur-Alzette, Luxembourg, L-4362 Luxembourg

**Keywords:** Biochemical networks, Computer modelling, Systems analysis

## Abstract

Crohn’s disease (CD) is associated with an ecological imbalance of the intestinal microbiota, consisting of hundreds of species. The underlying complexity as well as individual differences between patients contributes to the difficulty to define a standardized treatment. Computational modeling can systematically investigate metabolic interactions between gut microbes to unravel mechanistic insights. In this study, we integrated metagenomic data of CD patients and healthy controls with genome-scale metabolic models into personalized in silico microbiotas. We predicted short chain fatty acid (SFCA) levels for patients and controls, which were overall congruent with experimental findings. As an emergent property, low concentrations of SCFA were predicted for CD patients and the SCFA signatures were unique to each patient. Consequently, we suggest personalized dietary treatments that could improve each patient’s SCFA levels. The underlying modeling approach could aid clinical practice to find dietary treatment and guide recovery by rationally proposing food aliments.

## Computational modeling: Predicting diets for Crohn’s disease

Modeling of the human gut microbiota metabolism predicts that fiber-containing diets can improve the microbe metabolism in Crohn’s disease (CD). By integrating metagenomic data from human gut samples into personalized metabolic models, the fermentation profile of gut microbes and the differences between CD patients and healthy controls were assessed. To revert the fermentation differences between healthy and CD individuals, predicted beneficial metabolites were added to the *in silico* gut community models, with which the fermentation profile of CD patients could be improved. Taken together, these results can assist in the design of further experimental and clinical studies to aid CD patients with dietary supplementation.

## Introduction

The human gut microbiota is composed of thousand different bacterial species with a large functional diversity that surpasses the human gene pool.^1^ Health promoting functions of the gut microbiota include the breakdown of indigestible dietary fibers and production of short chain fatty acids (SCFA) utilized by the human host.^2,3^

Various human diseases, including inflammatory bowel disease (IBD), are associated with a loss of functional and taxonomic diversity of the gut microbiota.^1^ The main symptom of IBD is inflammation of the gut epithelium.^4^ IBD can be grouped into ulcerative colitis, primarily affecting the colon, and Crohn’s disease (CD), affecting various gastrointestinal sites. Non-invasive treatments for CD include the intake of antibiotics^5^ and steroid therapies.^6^ In addition, defined diet formulas are used to ease the symptoms of the disease.^7^ However, the success of these treatments varies between patients.^8^ Additionally, after remission, patients have difficulties in finding an appropriate diet and often experience relapse. Considering the human gut metabolism, it has been suggested that the diet reshapes the microbiota.^9^ Overall, the microbial diversity is decreased in CD patients. A shortage of SCFAs^10^ coincides with a decrease of fermenting Firmicutes bacteria.^11^ Microbial SCFAs have been recognized as important modulators of the immune system and as a nutrition source.^12^ Butyrate, for example, is taken up as an additional energy source by the host,^13^ contributes to epithelial barrier integrity,^14^ and stimulates the immune system.^15^ CD patients suffer from a low butyrate concentration,^16^ but its dietary supplementation can revert many of the IBD symptoms,^17^ highlighting the relevance of this particular SCFA in CD.

Given that the human gut microbiota is a complex microbial community with many different microbes that have varying metabolic potentials and substrate affinities,^18^ it becomes difficult to track the ecological interactions differing between CD patients and healthy individuals. Meta-omics approaches are generally used to characterize the microbiota and its metabolic potential.^19^ However, these top-down approaches do not provide mechanistic insights on the resilience of the microbiota and how perturbations, such as diets, may affect the system as a whole.

Bottom-up systems biology approaches can mechanistically describe biological systems and make relevant predictions. In particular, constraint-based reconstruction and analysis (COBRA) has been successfully applied to model the metabolism of different species and predict how perturbations affect the metabolic phenotype.^20,21^ Briefly, genome-scale metabolic reconstructions are represented by the complete set of biochemical reactions derived from a genome annotation and organism-specific literature in a stoichiometric accurate manner.^22^ Such high-quality manually-curated metabolic reconstructions are available for organisms from all three domains of life, such as *Escherichia coli*,^23^ yeast,^24^ and human (e.g.,^25,26^). Through the application of specific constraints (e.g., nutrient availability), the metabolic reconstructions can be converted into condition-specific models, which predict the reaction flux rates and growth yield under a given objective that is optimized using flux balance analysis (FBA).^20^ In a recent publication,^27^ we combined FBA with agent based modeling to simulate the ecology of microbial communities through the BacArena framework. Metabolic interactions emerge from the exchange of metabolites between species and the environment. These interactions can influence the metabolite concentration and the microbial community by inducing cross-feeding or resource competition. Such COBRA-based approaches provide a powerful mean to investigate mechanistic links in complex biological systems, such as the human gut microbiota.^28^

A recent study on pediatric CD sequenced the metagenomes of a North American cohort consisting of 26 healthy controls and 85 patients newly diagnosed with CD.^29^ In their study, the authors could distinguish two clusters of patients: A cluster of 57 patients, which had a microbiota composition similar to the healthy controls, and a cluster of 28 patients that had a distinguished dysbiotic microbiota. Compared to controls, these dysbiotic patients had a strongly differing functional and microbial abundance profile.

Here, we retrieved the original metagenomic data of the 26 healthy controls and 28 dysbiotic patients^29^ to simulate personalized in silico microbiotas with BacArena. We demonstrate that the simulated metabolic differences between patients and controls are congruent with experimental findings. We further show that predicted individual specific SCFA signatures are unique to each patient. Based on these results, we then predict personalized dietary treatments that would improve the SCFA concentrations of each patient. With this work, we demonstrate the added value of performing computational with integrating high-throughput data of individual microbiotas to predict mechanism-based personalized dietary intervention strategies for CD patients.

## Results

**Fig. 1:**
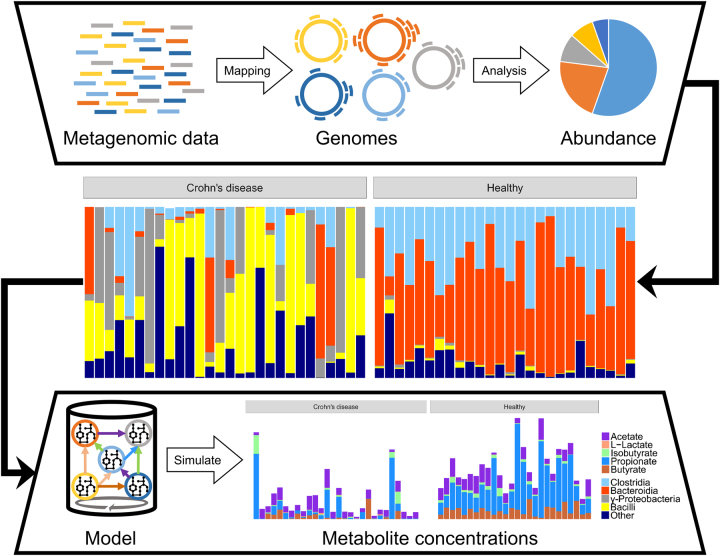
Computational framework used to create personalized metabolic models of gut microbial communities. Published metagenomic data were integrated into an in silico microbiota model for each CD patient and healthy control to simulate emergent metabolite concentrations

The aim of the present study was to predict in silico personalized dietary treatments for CD and investigate individual differences. We simulated personalized in silico microbiotas consisting of hundreds microbial metabolic models as defined by published metagenomic data of healthy controls and CD patients^29^ using a hybrid computational modeling approach,^27^ in which we combined FBA with agent based modeling to simulate the ecology of microbial communities through the BacArena framework. The predicted interactions can be used to gain further insight into metabolic differences that may contribute to CD and to propose modeling-assisted dietary intervention strategies for CD patients (Fig. 1). We describe differences between healthy controls and CD patients based on SCFAs as well as microbial abundances, which we validated with existing experimental knowledge. Individual differences within patients and controls were assessed to find individual specific SCFA signatures. Based on the individual microbiotas, personalized dietary treatments, such as supplementation of pectin and different glycans, were predicted to equilibrate the SCFA concentrations and promote healthier SCFA concentrations. Taken together, our work demonstrates the use of computational modeling to integrate existing high-throughput data of individual microbiotas and mechanistically predict personalized dietary treatments for CD.

### Microbial differences between healthy controls and CD patients

**Fig. 2:**
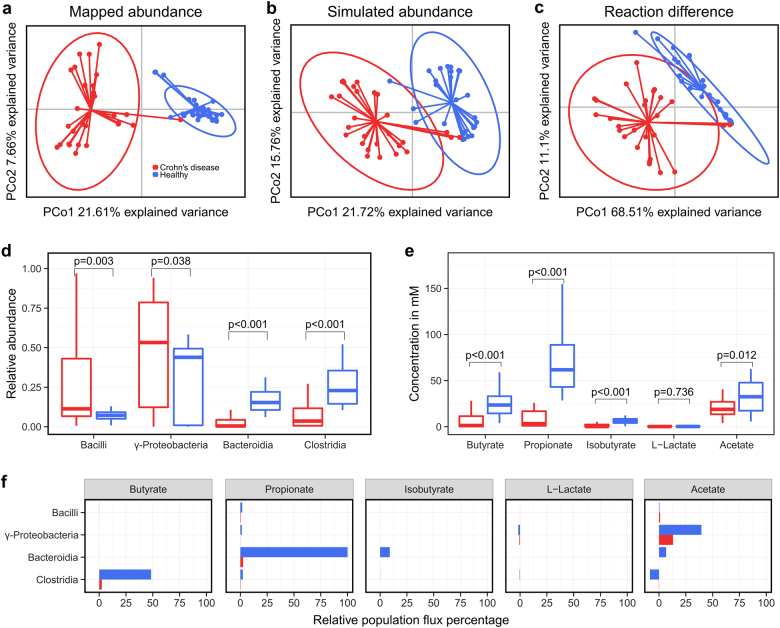
Metabolic and microbial group variability between healthy controls and Crohn’s disease patients. Similarities were assessed based on a principle coordinate analysis (PCoA) of the mapped abundance with Bray Curtis dissimilarity **a**, simulated abundances with Bray Curtis dissimilarity **b**, and reaction content with jaccard distance **c**. Based on the simulation, relative abundances **d** and metabolite concentrations of fermentation products **e** were compared (p-value determined by Wilcoxon rank-sum test). Microbial metabolic activities were displayed as the total population flux **f**

We ensured that our computational workflow (Fig. 1) would not alter the reported microbial differences between healthy controls and of dysbiotic CD patients.^29^ The workflow mapped the published metagenomic data of healthy controls and CD patients onto the genome sequences of the 773 gut microbial strains, for which metabolic reconstructions were available.^30^ On average, 283 +/− 240 of the 773 microbial strains were covered in the in silico microbiotas (Figure S1). Notably, the smallest microbiota contained only eight microbes, while the biggest had 713 of the 773 microbial strains. There were seven out of 54 in silico microbiotas that had less than 40 of the 773 microbes. While CD patients had generally less microbes, there were also some healthy controls with less than 40 microbes as well as CD patients with more than 600 microbes (Figure S1). Overall, the personalized in silico microbiota captured 73.5 +/− 16% of the relative microbial abundance from the original metagenomic reads. We could observe a clear separation of the healthy controls and CD patients based on microbial abundances (Fig. 2a), which was independent of the used similarity metrics (Figure S2). The most pronounced differences between healthy and CD individuals were due to significantly higher abundance of Bacilli and Gammaproteobacteria (*p* < 0.05, Wilcoxon rank-sum test) and significantly lower abundance of Bacteroidia and Clostridia (*p* < 0.001, Wilcoxon rank-sum test) in CD patients (Fig. 2d).

**Fig. 3:**
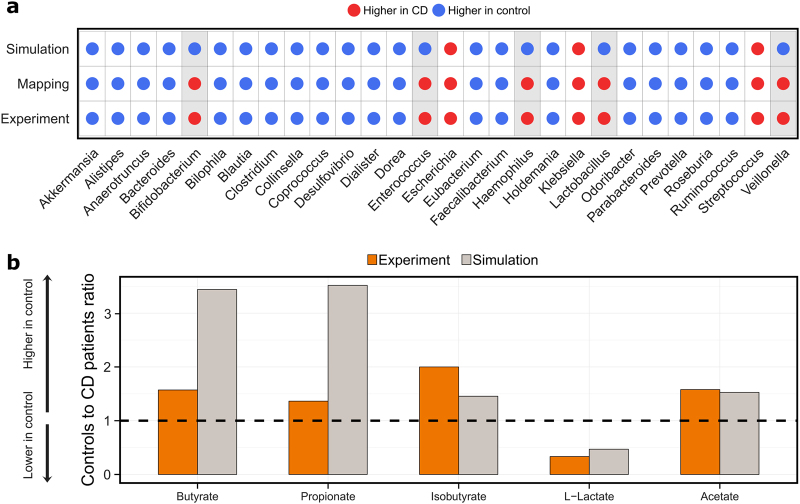
Qualitative comparison of simulation results with experimental values. Experimental relative abundances of microbial genera **a** were retrieved from the original study^29^ and compared with the abundances based on the mapped reads and simulations (*t* = 24 h). **b** Metabolite concentrations were retrieved from an independent experimental study^31^ and compared with the simulations (*t* = 24 h) based on the mean concentration ratios of healthy controls and CD patients

We then simulated the personalized in silico microbiota, inoculated with 500 microbes on a grid with 10,000 cells for 24 h, in the BacArena framework and analyzed whether the microbial abundances changed compared to the initial (metagenomic data driven) abundances. At the end of the simulation, the grid was populated by an average of 5902 +/− 1743 microbes (with an average grid occupation of 59 +/− 17%). Overall, the simulated abundances recapitulated the initial microbial differences, demonstrating that the in silico microbiotas were stable in BacArena (Fig. 2b). However, the abundance ratios of four out of 28 genera were higher in CD patients based on the simulated abundance, but lower based on the mapped data (Fig. 3a). In contrast, the mapped abundance data showed good agreement with the abundances reported in the original study (Fig. 3a, Figure S3). This discrepancy can be explained by the CD patients having a lower diversity of microbes, which led to a higher predicted abundance for the present genera.

Taken together, our workflow recapitulates the reported microbial differences between controls and CD patients.^29^ Furthermore, the simulation results of the personalized in silico microbiota in BacArena illustrate that these microbes can co-exist as stable microbial communities in silico.

### Emergent metabolic differences between healthy controls and CD patients

We investigated whether the difference in microbial abundance in the personalized in silico microbiotas also corresponded to differences in reaction content. In average, each personalized in silico microbiota consisted of 3,332,957 +/− 285,848 belonging to 3036 +/− 424 unique reactions. The presence and absence pattern of the unique reactions in the in silico microbiotas varied between individuals as well as between the two groups (Fig. 2c). Based on the reaction content, the first two principal components explained almost 80% of the variation in the data (Fig. 2c), and were mainly driven by the presence of transport reactions for fibers (Table S1). The observed reaction based separation is consistent with the aforementioned differences in microbial classes (Fig. 2d) and the distinct fiber metabolizing properties of Bacteroides.

SCFAs are important energy precursors and interact with the human immune system.^15^ We analyzed the secretion of SCFAs after 24 h by each personalized in silico microbiota to establish whether known microbiota-level differences in SCFA production could be reproduced by our modeling approach. The SCFAs butyrate, propionate, isobutyrate, and acetate were significantly lower in CD patients (*p* < 0.05, Wilcoxon rank-sum test, Fig. 2e). Only L-lactate levels were slightly higher in CD patients. To check for the validity of the simulated metabolite concentrations, we compared our results with an independent experimental study.^31^ The qualitative difference between CD patients and healthy controls were consistent with our simulations (Fig. 3b). However, the predicted concentrations of butyrate and propionate were three times higher in controls than in CD patients (Fig. 3b), which is much higher than the reported difference, likely due to the absence of the host cells in our model setup that can take up butyrate and propionate produced by the microbiota.^32^ Overall, our results confirm that the personalized in silico microbiotas recapitulate known differences in SCFA production levels in healthy and CD individuals.

An advantage of using computational modeling is that we can determine which microbes in the in silico microbiota caused the predicted differences in SCFA production. Therefore, we analyzed the summed uptake and secretion fluxes of each microbial class. We found that Clostridia were responsible for the production of 50% of the total butyrate, Bacteroidia produced almost 100% of the total propionate and about 10% of the total isobutyrate, Bacilli produced small quantities (<5% of the total concentration) of L-lactate, and Gammaproteobacteria produced almost 50% of the total acetate (Fig. 2f). Notably, in healthy controls, acetate was taken up by Clostridia illustrating cross-feeding between Gammaproteobacteria and Clostridia. These results demonstrated how changes in representatives of the main microbial classes can result in differences in SCFA production capabilities that differ significantly between healthy controls and CD patients.

### SCFA production profiles are patient-specific

**Fig. 4:**
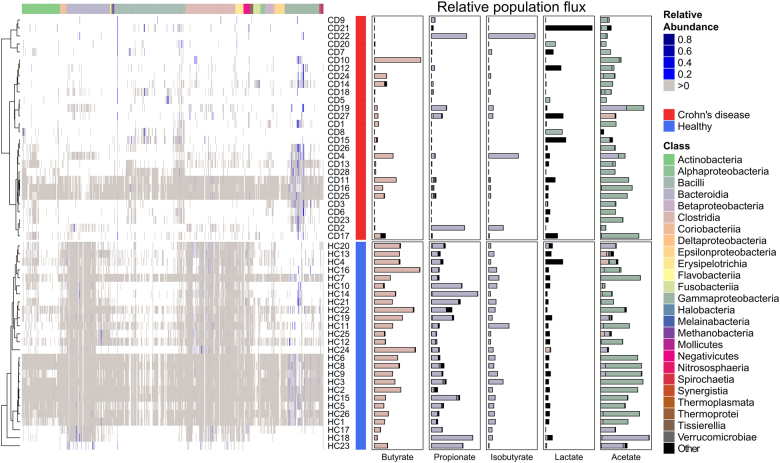
Individual variability between CD patients and healthy controls. The presence of different microbes is indicated by a gray color and the relative abundance by a blue color scale. Microbial taxa are based on the class level. Predicted metabolite concentrations are based on simulations. The microbial contribution to the concentrations are based on metabolic fluxes

The original metagenomic study^29^ reported the most distinct microbial differences between the healthy controls and the CD patients but also individual variability. Accordingly, the simulated relative microbial abundance also varied between the individuals (Fig. 4, left). We next investigated how much the predicted SFCA production varied between CD patients. Two (CD10, CD11) out of 28 CD patients had butyrate levels that were comparable to the mean of controls (mean concentration of 7.5 and 25.8 mM for CD and controls respectively). This could be explained by the higher activity of Clostridia species in these patients (Fig. 4, right). In three cases (CD2, CD4, CD22), the concentration of isobutyrate was higher in CD patients (Fig. 4) compared to the controls (mean concentration of 4.9 and 7.1 mM for CD and controls respectively). Two of these patients (CD2, CD22) had propionate levels comparable to the controls (mean concentration of 25 and 87.9 mM for CD and controls respectively), which is congruent with the high activity Bacteroides species (Fig. 4, right). Twelve out of the 28 patients showed increased L-lactate concentrations (mean concentration of 0.7 and 0.3 mM for CD and controls respectively), which can be attributed to the activity of Bacilli and other taxa (Fig. 4). Five patients (CD11, CD16, CD17, CD19, and CD25) showed acetate levels that were comparable to the controls (mean concentration of 21.1 and 32.2 mM for CD and controls respectively). This can be mostly attributed to the activity of Bacilli and Gammaproteobacteria (Fig. 4, right). Overall, these results indicated that every patient has a specific SCFA signature. This observation can be explained by the metabolic activity of the present microbiota, indicating that metabolic stimulation of the native CD microbiota may be able to revert some of the patient specific differences.

### Personalized dietary intervention strategies to normalize SCFA production capabilities of the personalized in silico microbiota

**Fig. 5:**
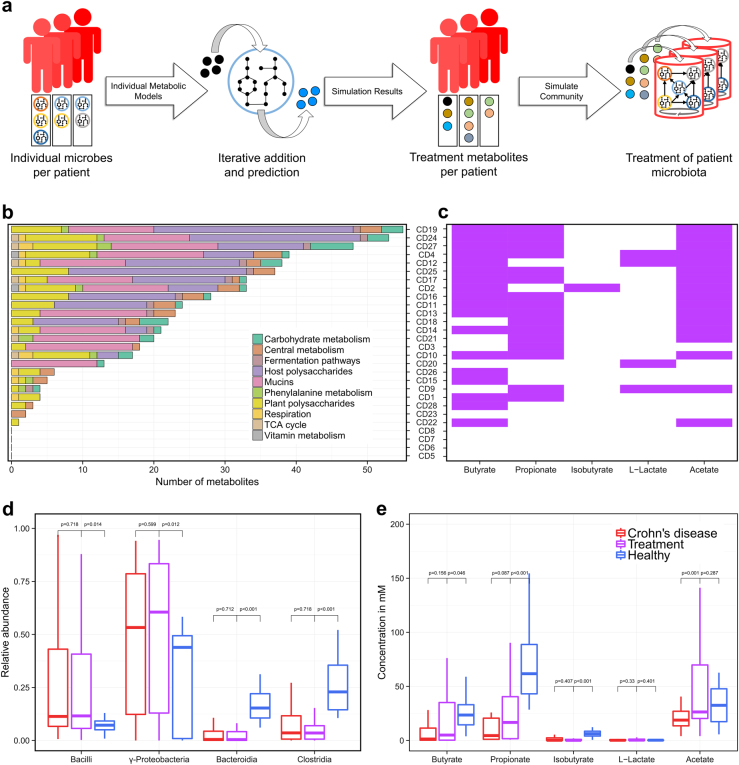
Individual treatment prediction for each CD patient. For the prediction of treatment metabolites **a**, single metabolic models of microbes for each patient were optimized for the production of the target metabolites with iterative dietary additions. **b** Shows broader categories of the predicted metabolites and **c** shows the response (metabolite increase of 25%) of each patient in purple. **d**, **e** show the relative abundance and metabolite concentrations

Defined dietary regimes are one possible treatment strategy for CD patients.^7^ However, the success of this treatment varies between CD patients.^2^ We investigated whether we could design personalized dietary interventions that would restore the SCFA production to levels commonly reported in healthy individuals. We approached this problem by predicting first whether increasing each dietary compound, present in the in silico rich diet, could individually lead to a healthier level of each of the five SCFAs in any microbial model present in a given patient (Fig. 5a). Interestingly, the number of the predicted dietary metabolites to be supplemented was specific for each patient and ranged between 1 and 55 metabolites (median of 19 metabolites) (Fig. 5b). For four out of the 28 CD patients, our described prediction approach did not identify any treatment. These four patients had a higher abundance of Gammaproteobacteria and Bacilli, while major SCFA producers were largely absent. For the remaining 24 CD patients, the most prominent category of the predicted metabolites were mucus glycans and glycosaminoglycans (Fig. 5b). In particular, pectin supplementation was predicted to be a good dietary supplement for 17 out of 24 CD patients (Figure S4). Other prevalent metabolites included various specific human produced mucus glycans and hepan/hyaluronan proteoglycan degradation products as well as plant-derived larch arabinogalactan, lavanbiose, and amylose.

We then added all of these identified metabolites to each of the personalized in silico microbiota to ensure that the community could also produce healthier SCFA levels. Each in silico microbiota was simulated for 24 h in the supplemented diets. The success of the in silico dietary interventions varied between patients (Fig. 5c). Overall, the most successful metabolite level restoration was obtained for butyrate, propionate, and acetate, whereas the in silico treatment was less successful for isobutyrate and L-lactate (Fig. 5c, e). The in silico treatments had only small effects on the relative species abundances (Fig. 5d) due to the dysbiotic patients lacking the relevant microbes found in healthy individuals. Therefore, our results showed quantitatively improved levels of SCFAs on the individual patient level as well as on the differences between patients and healthy controls.

## Discussion

We created personalized in silico microbiota of healthy controls and CD patients by integrating metagenomic data into a bottom-up systems biology framework (Fig. 1). Recent approaches have successfully integrated metagenomic data to model the ecological dynamics of the human gut microbiota^33^ but lack the metabolic aspect, which plays an important role for human health and disease.^34^ Therefore, the added benefit of our modeling approach is combining metabolism with ecology to investigate the metabolic activity of the gut microbiota.

To find strong differences between CD patients and healthy controls, we selected data of dysbiotic patients, defined by their microbial distance to healthy controls.^29^ Expectedly, we could reproduce the microbial differences originally reported in the study (Fig. 2a). Moreover, our reference based assessment was consistent with the reference independent analysis in the original study (Fig. 3a), which further demonstrates that the set of 773 AGORA microbes capture the most common human gut microbes.^30^ When comparing the abundance of specific genera (Fig. 3a), the community simulations predict differing ratios for four out of 28 genera, indicating a minor variability in the simulations that did not affect the overall differences (Fig. 2b). The main microbial differences between CD patients and healthy controls can be attributed to a decreased abundance of Bacteroidia and Clostridia as well as an increased abundance of Bacilli and Gammaproteobacteria in CD patients (Fig. 2d), which was in accordance with an independent experimental study^35^ and characteristic for a dysbiotic microbiota, as a specific case of CD.^29^ This approach thus allows us to address fundamental questions in CD dysbiosis and how the microbiota can shape metabolite concentrations, which is less understood so far.

The simulated SCFA concentrations represent emergent properties of our models that could not be achieved by the metagenomic data alone. As shown in our previous study,^27^ the modeling approach can aid in the understanding of SCFA production of gut microbes as validated by experimentally determined in vitro concentrations. Therefore, we could simulate clinical relevant metabolite concentrations, known to be differentially regulated in CD.^31^ Interestingly, we could detect higher concentrations of acetate, propionate, butyrate, and isobutyrate as well as a lower concentration of L-Lactate in controls (Fig. 2e). Based on the quantitative ratios between controls and patients, butyrate and propionate were higher in our simulations than in the experimental literature^31^ (Fig. 3b). This apparent discrepancy could be explained by the uptake of butyrate and propionate by the host,^2^ which we did not include, highlighting a limitation of our current modeling approach. SCFAs, in general, have been associated with healthy gut functions, such as energy conversion of the host as well as immune stimulation.^12^ Butyrate, in particular, mediates the immune system^15^ and influences the tight junctions between epithelial cells.^14^ Moreover, butyrate, as well as propionate, are carbon sources for colonocytes.^36,37^ Taken together, the added value of our modeling approach is that we can predict these qualitative changes in SCFA levels, which we can attribute to specific microbial metabolic activity.

We identified which microbes are responsible for the production of the SCFA (Fig. 2f). Clostridia produced mainly butyrate explaining its lower concentration in CD patients (Fig. 2e), who had generally lower Clostridia abundances (Fig. 2d). The Clostridia, Faecalibacterium, and Roseburia, are known to be the main butyrate producers,^38^ which were decreased in abundance in CD patients (Fig. 3b). We identified new metabolic interaction patterns, such as the consumption of acetate by Clostridia (Fig. 2f). In vitro experiments have demonstrated cross-feeding interactions between Clostridia and Bifidobacterium species.^39^ These metabolic interactions link microbes with metabolites and demonstrate that we capture in silico the gut microbiota as a whole.

Our personalized in silico microbiota modeling approach permitted the investigation of individual differences between CD patients and healthy controls (Fig. 4). Overall, we found that healthy controls have a higher microbial diversity than CD patients, which is also confirmed by experimental knowledge.^11^ Consequently, controls have more comparable SCFA levels (Fig. 4), indicating metabolic consistency through functional redundancy.^40^ Based on the individual SCFA variability, one could speculate that the microbiota of CD patients can compensate some metabolic differences but lacked functional redundancy and diversity to consistently establish a healthy SCFA signature (Fig. 4). This observation further underlines the importance of a diverse microbiota, which can complement potential metabolic shortcomings between microbes. Further studies could investigate the importance of keystone species in this context, which have a low abundance but high metabolic activity and thus ecological relevance.^41^

In our in silico treatment predictions, we take the individual factors into account by designing dietary supplements compensating individual differences (Fig. 5a). Most of the predicted treatment metabolites were mucus glycans, glycosaminoglycans, and plant polysaccharides (Fig. 5b), further indicating that fibers are relevant in shaping the gut microbiota metabolism.^42,43^ Particularly, pectin was predicted as a potential treatment for the majority of patients, which further underlines the dietary relevance of this compound.^42^ Plant fibers and host glycans influence the gut microbiota by stimulating Clostridia and Bacteroidia species,^44^ which produce butyrate and propionate, respectively (Fig. 2f). Interestingly, the predicted metabolite cocktails were different for each patient (Fig. 5b, Figure S4). In clinical practice, a standard dietary formula in form of exclusive enteral nutrition is used to treat patients with CD.^7^ However, not every patient responds equally well to different diet formulations, which vary in their fiber content.^45^ Current knowledge is limited when defining personalized diets because of the complexity of the human gut microbiota and its intricate response to different diets. Some patients suffer from relapse when switching to a normal diet after successful remission.^46^ In such cases, our modeling-based predictions could give new directions on aliments based on a patient’s microbiota. Furthermore, using computational modeling in conjunction with metagenomic data, the dietary treatment could be readily redefined and adjusted to match the patient’s need. To our knowledge, such modeling-guided dietary treatment approach is not available yet for CD patients. As a next step, our predictions need to be validated in a nutritional trial. Then, our systematic approach to defining personalized nutrition therapies could guide clinicians and nutritionists in designing new, personalized diet-based treatments.

Testing our in silico dietary treatments on each patient’s’ microbiota, we found an improvement in SCFA levels. Butyrate, propionate, and acetate showed an overall success in shifting levels, while isobutyrate and L-lactate were less successful (Fig. 5c, e), since these SCFAs only had a minor difference between controls and patients (Fig. 2e). The overall microbe abundance did also not shift significantly in the treatment condition (Fig. 5d), because patients had a lower diversity from the start (Fig. 4, Figure S5) and could not acquire the necessary microbes to compensate their abundance profile. In this context, the integrated microbial abundances might have been in an ecological steady state while sampling and therefore, they did not respond in the population dynamics analysis. Further studies could simulate the effect of adding specific microbe models as a treatment, which could be integrated in our framework. Furthermore, human metabolism could be integrated with the in silico microbiota to investigate the reciprocal effect on the host, and, for instance, the effect of colorectal cancer cells that might be affected by butyrate concentrations.^47^

Several studies emphasize the need for computational models to discover mechanisms for microbiota associated diseases.^28,48–51^ Our approach introduces metabolism as an additional emergent property of the microbiota yielding new mechanistic insight of SCFA production by microbial communities. Our results indicate an individual specific dietary response of the gut microbiota, which is not generalizable for all CD patients. In subsequent studies, one could integrate further patient metagenomic data with our modeling framework to predict potential dietary treatments, which yet have to be validated in a clinical setting. An extension for possible treatment strategies includes the simulation of probiotics and fecal transplantation. In fact, our model could be used as an additional workflow for donor optimization of fecal transplantation.^52^ Furthermore, the computational modeling approach that we presented is not limited to the application of CD but can be applied to any metagenomic data set. Taken together, we present a powerful, expandable, versatile computational modeling approach that permits to yield insight into metabolic interactions emerging from personalized metagenomic data.

## Methods

### Retrieval of metagenomic data and pre-processing

Paired-end Illumina raw reads of a study on early onset CD patients and healthy controls of a North American cohort^29^ were retrieved from NCBI SRA under the accession: SRP057027. Based on the studies’ definition of healthy and dysbiotic individual microbiotas,^29^ the samples were selected to a smaller subset of 26 healthy controls and 28 CD patients to capture the most pronounced differences in the individual microbial communities. Furthermore, only the first measured time point was selected to represent newly diagnosed and yet untreated microbiotas. The reads were quality trimmed using Trimmomatic^53^ with default parameters for paired-end Illumina sequences. To remove human contaminant sequences, the reads which were still paired were mapped with default parameters using the software BWA^54^ to the human genome version 38 (http://www.ncbi.nlm.nih.gov/projects/genome/assembly/grc/).

### Metagenomic mapping and abundance estimation

Using BWA,^54^ the pre-processed reads were mapped with default parameters onto a reference set of 773 genomes, which were selected according to a previous study.^30^ Before mapping, the reference genomes of these organisms were combined into one file where each genome is represented as a chromosome. To filter out cross-mapped reads (reads mapped to multiple positions), samtools^55^ was used to discard mapped reads with a low-quality score. The coverage per genome (number of mapped reads normalized by genome size) was calculated using samtools. To reduce the number of false positives, we set a threshold of at least 1% genome coverage for each microbe in each human individual. In accordance to another pipeline,^56^ the resulting coverages were normalized for each individual to obtain the relative microbe abundances.

### Microbial metabolic reconstructions

We retrieved published gut microbial metabolic reconstruction^30^ from http://vmh.life. These microbes have been chosen according to their prevalence in the human gut and the availability of a genome sequences, and they have been extensively curated based on available physiological and biochemical data.^30^

### Analysis of mapped abundance and reaction differences

The mapped microbial abundances for each individual were compared by computing the Bray-Curtis similarity and subsequent visualization with principal coordinate analysis (PCoA) using the R package vegan.^57^ The unique reaction set of personalized in silico microbiotas was determined by taking the union of all present microbe reactions retrieved from the corresponding metabolic model^30^ of each microbe. PCoA was performed on the metabolic distance between each individual’s reaction set similar to.^30^

### Setup, integration, and simulation of the personalized microbiota models

The next step is to integrate the abundance information into a personalized in silico microbiota for each person. Therefore, we used a previously established R package for community modeling,^27^ which represents bacteria as individuals in a grid environment that can exchange metabolites by secretion and uptake. Individual optimizations were carried out using the microbial biomass as an objective. Consequently, the observed concentrations of metabolites, in particular SCFA, are a product of the individual microbial energy metabolism. The dimensions of the two-dimensional quadratic environment were set 0.025 cm^2^ with 100 grid cells per side length. This resulted in 10,000 grid cells that could be potentially occupied by the microbes. To allow space for the in silico microbial community to grow, 500 microbes were initially added to the grid environment. The relative microbial abundances were used to scale the number of microbes to be added per species (e.g., if one species has a relative abundance of 0.01, 5 microbes were added for this species). In case the calculated number of microbes resulted in decimal places, we rounded the final number to the next highest integer. Hence, all microbes that were detected as present in the samples, were included and had an initial microbiota size ranging between 505 and 1109 microbe individuals. All possible metabolites (union of metabolites that can be taken up by each microbe) were added to the environment with a minimal concentration of 0.2 µM to provide a rich medium that is consistent between individuals. Therefore, metabolite concentrations that emerge from the simulations can be specifically attributed to the microbiota of each individual.

Once the in silico microbiota for each CD patient and healthy control have been setup in BacArena, the growth of each microbial model in the microbiota was sequentially for each time step. A total of 24 time steps were simulated, one per hour, corresponding to an overall simulation time of 24 h. To reduce the complexity of the model, we simulated a well-mixed environment in which metabolite concentrations are uniformly distributed and microbes move randomly.

The R package Sybil^58^ was used for constraint-based modeling with ILOG CPLEX as a linear programming solver.

### Analysis of simulation results

After the simulation, each personalized in silico microbiota was primarily analyzed in terms of the microbe abundance and metabolite concentrations. Since the simulations include temporal dynamics with different time points, we chose the last time point (24 h) for our analysis and comparison between individuals. This allowed the in silico microbial communities enough time to consume and produce metabolites, and to reach a steady state. The microbial abundances were determined by assessing the number of microbes in each personalized in silico microbiota. The vector of microbial abundances was then compared by computing the Bray-Curtis similarity with PCoA visualization. Abundances of specific taxa were calculated by summing up the relative abundances of each corresponding representative. The abundances of the most differing taxa were tested for significant differences between healthy controls and CD patients with the Wilcoxon rank-sum test^59^ (26 controls and 28 CD patients) implemented in R.

Metabolite concentrations were determined by their molar concentration in the environment at the end of the simulation (*t* = 24 h). The concentration of the most relevant metabolites, butyrate, propionate, isobutyrate, L-lactate, and acetate, were assessed and tested for significant differences between the personalized in silico microbiota of healthy controls and of CD patients using the Wilcoxon rank-sum test. To investigate the influence of each microbial taxa on the metabolite concentrations, we further evaluated the metabolic fluxes of each microbe in the personalized in silico microbiota. For each taxa, the reaction fluxes in all corresponding microbes were summed up.

### Definition of personalized dietary treatments

After identifying the metabolic signatures influencing the differences between healthy controls and CD patients, we predicted metabolites that could revert these differences:

According to their presence in each personalized in silico microbiota, the set of microbes was selectively analyzed for every individual. Each personalized in silico microbiota was then simulated in a rich medium containing all possible metabolite with flux uptake constraints of 1 mmol gDW^−1^ h^−1^ and the biomass as well as the production of SCFAs (butyrate, propionate, isobutyrate, L-lactate, acetate) were optimized separately. To enhance the growth of beneficial bacteria, we selected metabolites based on the ability of the CD low abundant microbes (e.g., Clostridia, Bacteroides) to uptake these nutrients over the CD high abundant microbes (e.g., Gammaproteobacteria, Bacilli). We then added the selected metabolites iteratively to the in silico medium with a maximal flux uptake constraint of 1000 mmol gDW^−1^ h^−1^ to investigate whether the SCFAs increased or decreased. Based on these simulations, the added metabolites which had a positive effect (recovering metabolite production to healthy levels) were then collected and used as the personalized dietary treatment for each individual.

We tested the effect of the treatment on the personalized in silico microbiota of CD patients by adding a 100 times higher concentration of the predicted treatment metabolites to the in silico rich diet containing 0.2 µM for each metabolite. The personalized in silico microbiota simulations and analyses were then carried out as described above.

### Data availability

The scripts to construct and simulate the individual specific microbiota models as well as the analysis scripts are available on GitHub: https://github.com/ThieleLab/CodeBase

## Electronic supplementary material

Table S1

Table S2

Supplemental figure legends

## Electronic supplementary material

**Supplementary information** accompanies the paper on the *npj Systems Biology and Applications* website (10.1038/s41540-018-0063-2).
