# Supplementary material for: From metagenomic data to personalized in silico microbiotas: predicting dietary supplements for Crohn’s disease
Source: NPJ Syst Biol Appl. 2018 Aug 1;4:27. doi: 10.1038/s41540-018-0063-2 (PMC6068170; doi:10.1038/s41540-018-0063-2)
Supplement: Supplementary file 3 — Supplemental figure legends [file 41540_2018_63_MOESM3_ESM.docx]

| **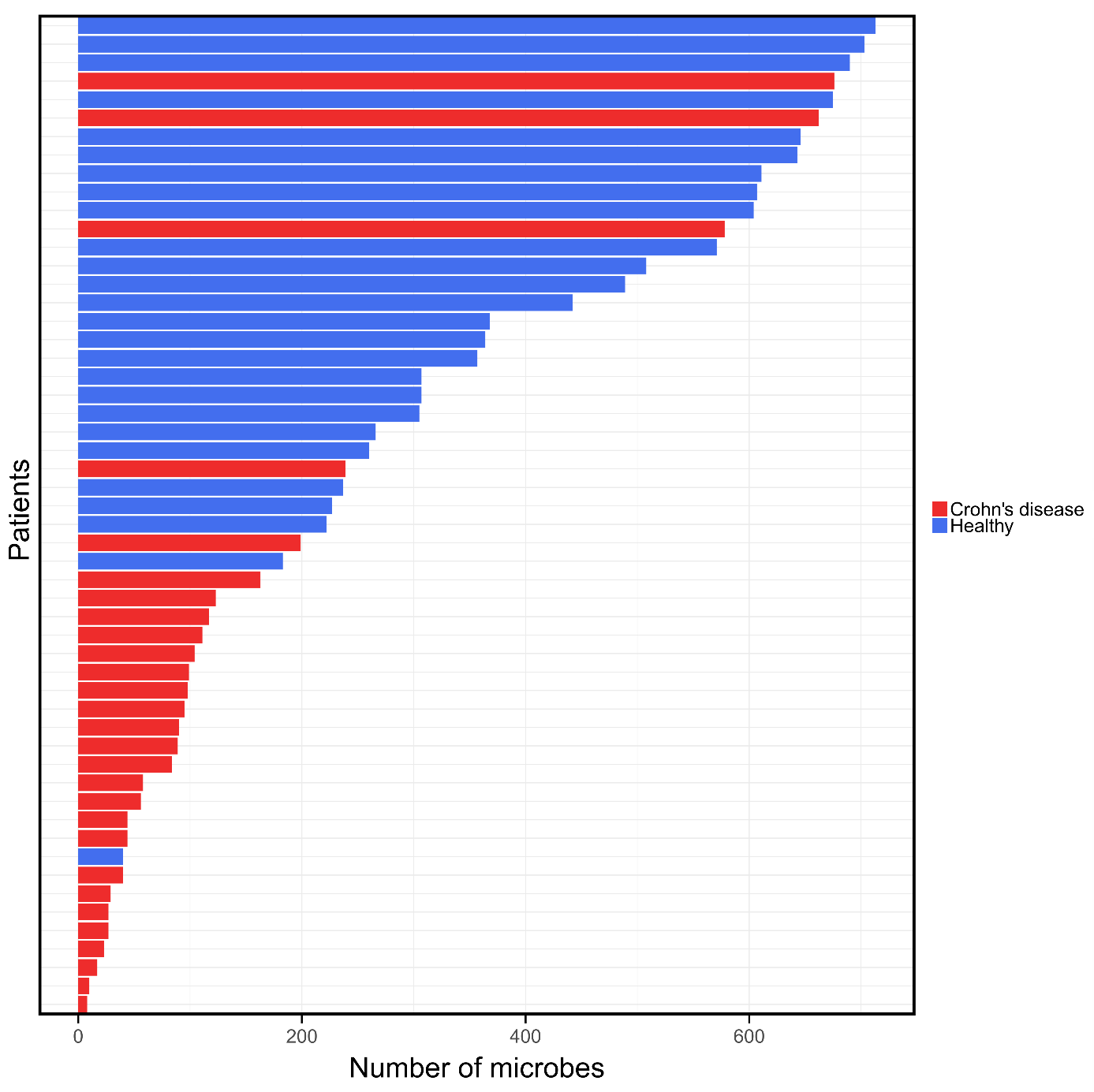** |
| --- |
| **Figure S1.** Number of microbes that were detected to be present for each Crohn’s disease patient and healthy control. |

| **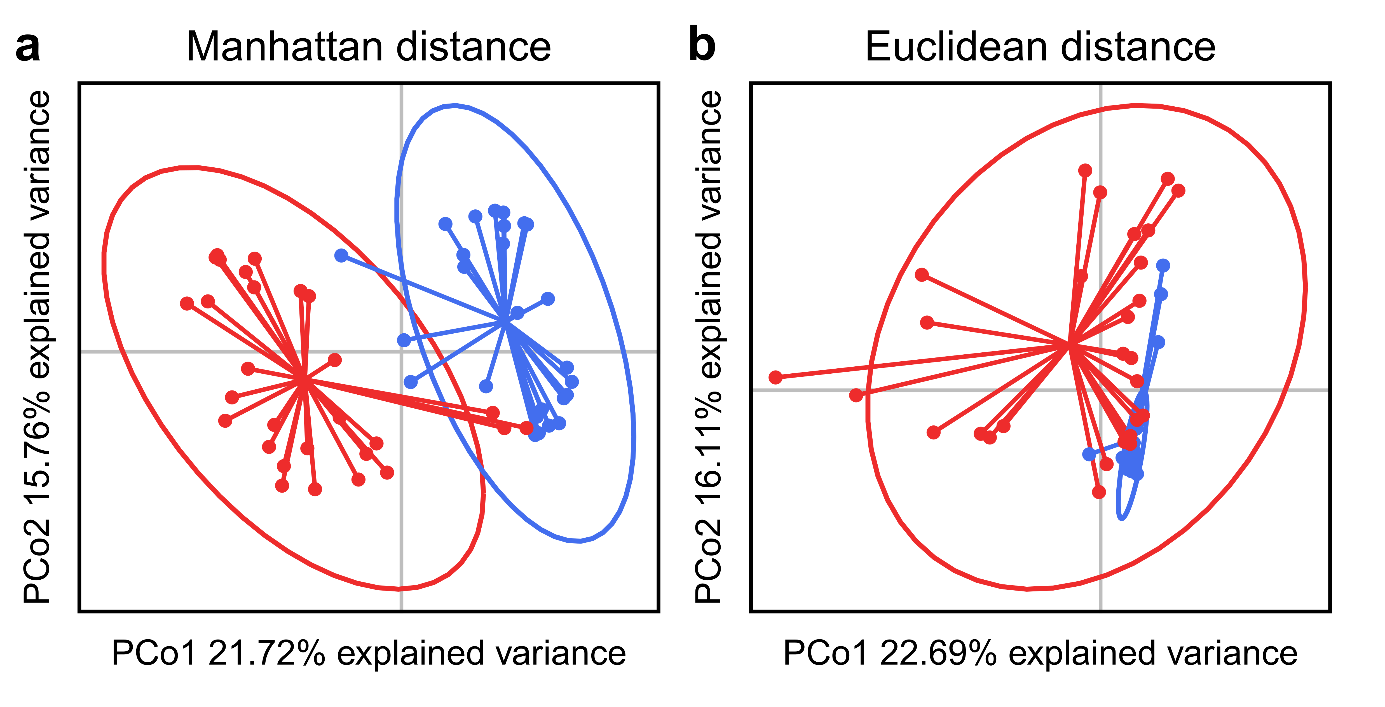** |
| --- |
| **Figure S2.** Similarities between healthy controls and Crohn’s disease patients assessed based on a principle coordinate analysis (PCoA) of the mapped abundance with Manhattan **(a)** and Euclidean distance **(b)**. |

| **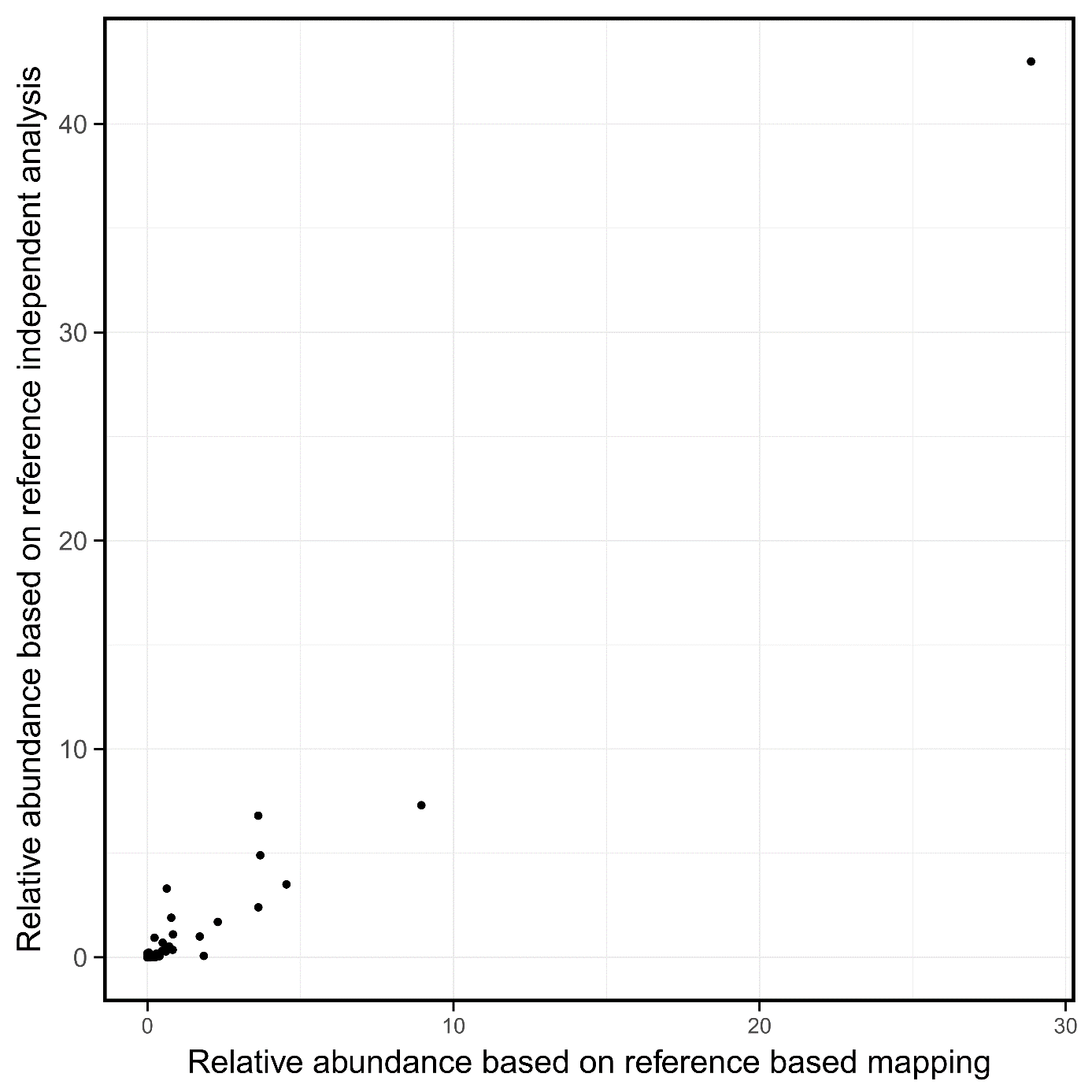** |
| --- |
| **Figure S3.** Quantitative comparison of mapped microbe abundance values compared to the relative abundance of genera retrieved from the original study. |

| **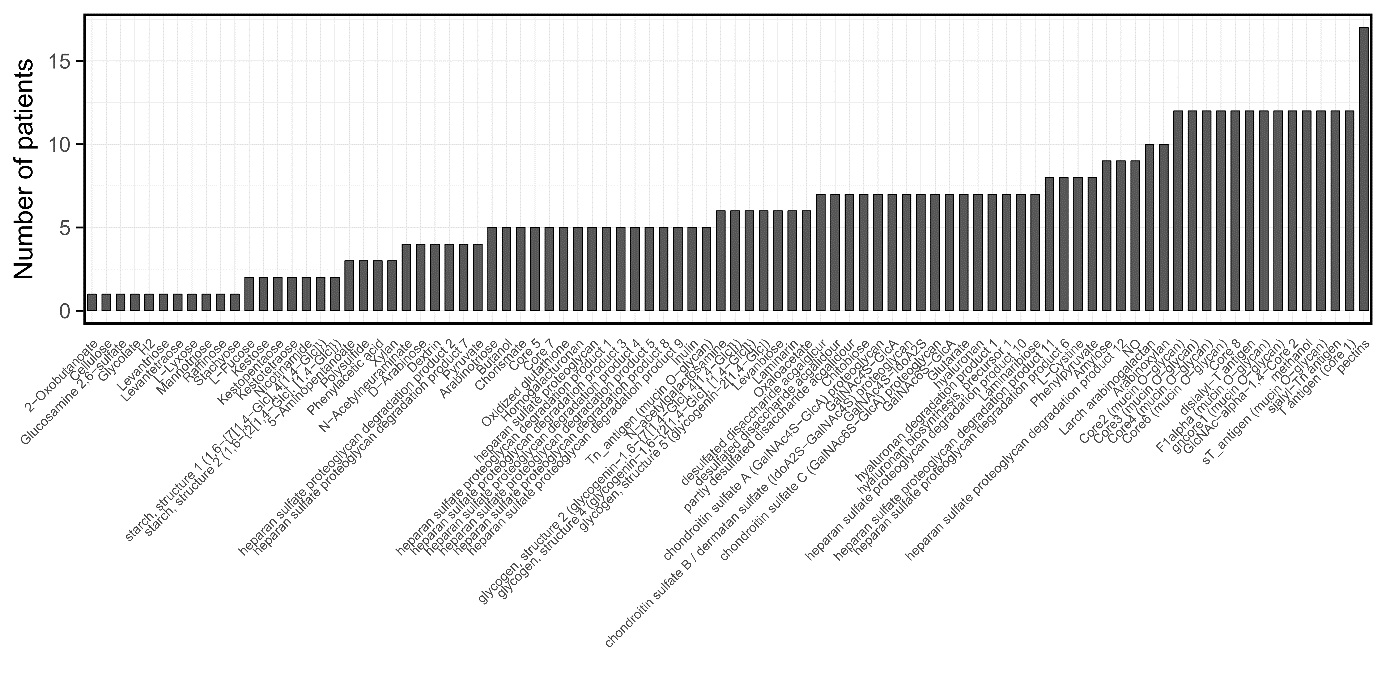** |
| --- |
| **Figure S4.** Predicted metabolites used for *in silico* treatment of each patient. The number of patients is given in which the metabolite addition had a beneficial effect. |

| **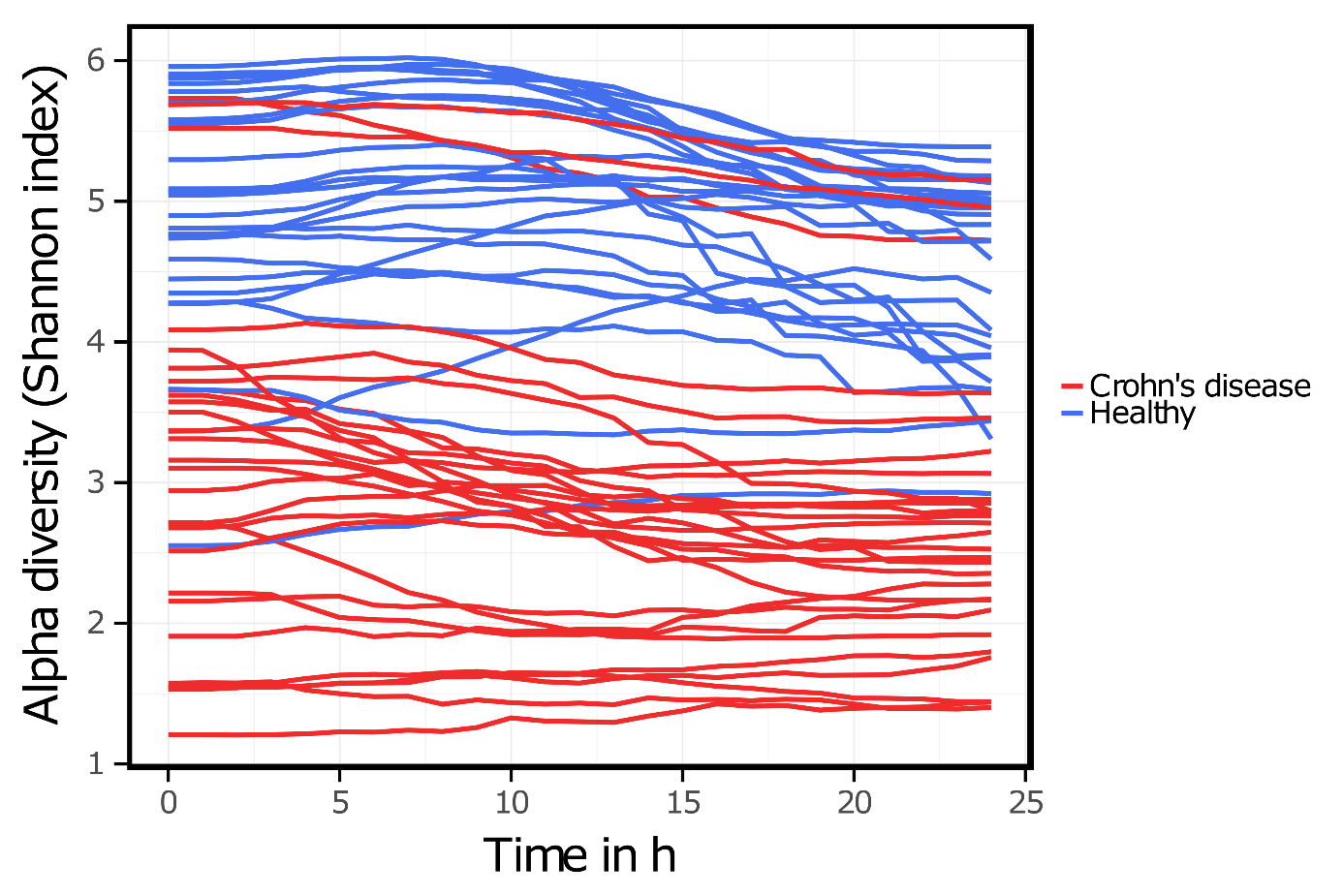** |
| --- |
| **Figure S5.** Alpha diversity (determined by the Shannon index) of the individual microbiotas throughout the complete simulated time steps. |
